# The Impact of Intranasal Oxytocin on Attention to Social Emotional Stimuli in Patients with Anorexia Nervosa: A Double Blind within-Subject Cross-over Experiment

**DOI:** 10.1371/journal.pone.0090721

**Published:** 2014-03-06

**Authors:** Youl-Ri Kim, Chan-Hyung Kim, Jin Hong Park, Jimin Pyo, Janet Treasure

**Affiliations:** 1 Department of Neuropsychiatry, Seoul Paik Hospital, Inje University, Seoul, Republic of Korea; 2 Department of Psychiatry, Severance Mental Hospital, Yonsei University College of Medicine, Gyeonggi Do, South Korea; 3 Department of Psychology, Carleton College, Northfield, Minnesota, United States of America; 4 Section of Eating Disorders, Department of Psychological Medicine, King's College London, Institute of Psychiatry, London, United Kingdom; Royal Holloway, University of London, United Kingdom

## Abstract

**Background and aim:**

Social factors may be of importance causally and act as maintenance factors in patients with anorexia nervosa. Oxytocin is a neuromodulatory hormone involved in social emotional processing associated with attentional processes. This study aimed to examine the impact of oxytocin on attentional processes to social faces representing anger, disgust, and happiness in patients with anorexia nervosa.

**Method:**

A double-blind, placebo-controlled within-subject crossover design was used. Intranasal oxytocin or placebo followed by a visual probe detection task with faces depicting anger, disgust, and happiness was administered to 64 female subjects: 31 patients with anorexia nervosa and 33 control students.

**Results:**

Attentional bias to the disgust stimuli was observed in both groups under the placebo condition. The attentional bias to disgust was reduced under the oxytocin condition (a moderate effect in the patient group). Avoidance of angry faces was observed in the patient group under the placebo condition and vigilance was observed in the healthy comparison group; both of these information processing responses were moderated by oxytocin producing an increase in vigilance in the patients. Happy/smiling faces did not elicit an attentional response in controls or the patients under either the placebo or oxytocin conditions.

**Conclusion:**

Oxytocin attenuated attentional vigilance to disgust in patients with anorexia nervosa and healthy controls. On the other hand, oxytocin changed the response to angry faces from avoidance to vigilance in patients but reduced vigilance to anger in healthy controls. We conclude that patients with anorexia nervosa appear to use different strategies/circuits to emotionally process anger from their healthy counterparts.

## Introduction

Anorexia nervosa (AN) is characterized by problems with eating, weight, and shape. Shape and weight concerns often involve social contagion and social evaluation [1] and fat talk, teasing, and bullying often trigger the onset of the illness [2], [3]. In addition, social difficulties may occur early in the development. Gillberg (1992) suggested an association between developmental traits such as low empathy and a later risk of developing an eating disorder [4]. Patients with eating disorders, particularly those with AN, start showing signs of social difficulties before the actual onset of the illness [5], and social problems at age 8 strongly predict eating disorder onset at age 14 [6]. These social difficulties become more exaggerated during the illness leading to interpersonal problems and/or isolation [7]. A medical practitioner elaborated on the lack of social skills in AN by saying that if she had to use one word to describe what she saw in her patients with AN, it would be “isolation” [8]. Loneliness and social exclusion heighten sensitivity to social threats, increase depressive symptomatology, and aggravate many of the psycho-physiological consequences of stress and chronic inflammation [9]. Social exclusion in primates is associated with a variety of critical metabolic consequences, including reduced levels of oxytocin [10]. These studies suggest that social factors may be contributing to the causal and the maintenance factors of AN [7], [11].

The social processing problems in patients with eating disorders have been synthesized in recent systematic reviews, and impairments in emotional recognition, social hierarchy, and theory of mind have been found [12]–[15]. Additionally, patients with AN display less facial emotional expression, to either emotionally salient films or to challenging situations, while showing high levels of emotion on self-report measures [16], [17]. Some of these difficulties in social and emotional functioning include problems with attentional processing. Patients with AN (in both acute and recovered states) have an attentional bias towards rejecting criticism and away from accepting positive facial expressions [18]. This observation suggests that patients with AN are sensitized to socially evaluative threats and are less responsive to social rewards.

Oxytocin is an important neuromodulatory hormone involved in social emotional processing, particularly in association with attentional processes. Oxytocin increases the sensitivity to detect emotional expressions, which is associated with recruitment of attentional resources, as evidenced by pupil dilation [19]. Oxytocin also enhances evaluative processing of both explicit and hidden positive and negative facial expressions [20]. Oxytocin modulates the attention paid to threat and reward. Rhesus monkeys, when given oxytocin, pay less attention to threatening facial expressions [21]. In humans, oxytocin reduces eye gaze to angry expressions [22], and in healthy females, oxytocin increases amygdala reactivity to both social and non-social threats [23]. In contrast, in women with borderline personality disorder, oxytocin decreases early eye gaze and amygdala reactivity to social threats [24]. A study using the dot probe paradigm found an avoidant pattern to threat but this pattern was normalized when oxytocin was administered [25]. Oxytocin also increases eye gaze to happy expressions in human [22], [26] and early attention to happy faces in young men [27]. However other studies have found that oxytocin does not influence detection of positive emotion (happiness) [28], [29]. In summary, when the analyses were restricted to facial expression types, significant effects of oxytocin on recognition accuracy were specifically found for recognition of happy and fear faces [30]. Although there are individual and perhaps gender differences in attentional processes (avoidance vs. vigilance), the overall effect of oxytocin is that it decreases attention to threat and increases attention to happiness.

It is possible that changes in oxytocin function may account for some of the anomalies in social cognition in patients with AN. A recent review synthesized evidence for abnormal oxytocin functioning in patients with AN [31]. Cerebrospinal fluid levels of oxytocin decrease during the starvation phase of AN [32]–[34]. Nocturnal serum oxytocin levels in patients with AN are also reduced [35]. Lawson et al. [34] reported that the release of oxytocin increases in response to a meal in the acute state of AN and decreases after recovery. Abnormalities in oxytocin secretion are correlated with severity of the disordered eating psychopathology and with the level of activation of trait anomalies in oxytocin function in the brain circuitry in patients with AN. Therefore, it is of interest to examine whether oxytocin moderates some of the social processing problems in patients with AN.

The aim of this study was to examine whether oxytocin impacts on social attentional processes in patients with AN. Our first hypothesis, based on previous studies using the dot probe paradigm, is that patients with AN would have vigilance towards threatening cues and away from rewarding stimuli. Our second hypothesis is that oxytocin would attenuates the selective attention towards negative social cues.

## Methods

### Participants

Sixty-four women (31 patients with AN and 33 healthy university students) took part in this double-blind, placebo-controlled cross-over study. The first participant entered the study on August 16, 2012, and the last participant was examined on November 30, 2013. The patients with AN were recruited at the Eating Disorders Clinic of Seoul Paik Hospital, Seoul, South Korea. As this study aimed to examine broadly whether oxytocin might be of benefit in the short term for patients with AN, patients were recruited at various stages and phase of illness both from an outpatient clinic (n = 13) and an inpatient ward (n = 18). All patients who volunteered to participate were screened during the early phase of treatment. All of them were in an active stage of illness, and none of them were in the weight-recovered stage. The diagnosis of AN was confirmed by the Structured Clinical Interview from the Diagnostic and Statistical Manual of Mental Disorders, Fourth Edition [36]. Exclusion criteria for patients were: active substance use disorder, diagnosis of a psychotic disorder (schizophrenia, schizoaffective, psychosis not otherwise specified), diagnosis of autism or Asperger's syndrome. All other comorbid diagnoses were allowed. Patients taking any psychiatric medications influencing attentional bias were excluded [37], although three patients who had been taking a stable low dose (20 mg/d) of fluoxetine for several weeks were included. The participants in the comparison group were undergraduate or graduate students who had responded to an advertisement posted in the psychology department at a women's university in Seoul, South Korea. The inclusion criteria were: healthy females without a history of medical or psychiatric illnesses and a minimum of 18 years old. All subjects were postpubertal, nonsmokers, heterosexual, nulliparous, and were not taking any medications (including the contraceptive pill). Exclusion criteria included a self-reported history of major depression, bipolar, panic, or psychotic disorders, substance dependence, epilepsy, eating disorders, autism spectrum disorder, or traumatic brain injury.

The Korean version of the Wechsler Adult Intelligence Scale was used to measure IQ. Healthy participants were tested during the follicular phase of their menstrual cycle. None of the patients were menstruating. Compensation was provided for travelling expenses and time. This study protocol was approved by both the Korean Food and Drug Association Institutional Review Board (12061) and the Institutional Review Board of Seoul Paik Hospital (IIT-2012-096). All participants provided written informed consent prior to participating in the study. Consent was provided by both the patients and their guardians in the case of 16-year-old patients.

### Oxytocin preparation

The intranasal oxytocin spray was formulated by *JW* Pharmaceuticals (Seoul, South Korea) from oxytocin powder (Hemmo Pharmaceuticals, Mumbai, India). The solution was prepared by combining 35.2 mg oxytocin (568 U) with 300 mL 0.9% sodium chloride solution, and adjusting the pH to 4.01 with 10× diluted acetic acid. The placebo spray (pH 4.01) was formulated with 0.9% sodium chloride solution and acetic acid, but without the peptide. The filtered and sterilized solution was sealed in individual vials (1.5 mL each) and stored frozen. On the day of use, the vials were thawed and kept in a refrigerator (4°C) until required. A clinician prepared the nasal spray by transferring oxytocin or the placebo from the vial into a nebulizer.

### Oxytocin or placebo administration

Oxytocin and the placebo were administered intranasally 4–7 days apart 45 min before the neuropsychological tasks. We chose a dose of 40 IU of oxytocin based on a recent review reporting that cumulative evidence from clinical trials shows that short-term use of intranasal oxytocin administered to both male and female humans in dosages up to 40 IU (per dose) results in no detectable subjective adverse effects in a controlled research setting [38]. Each participant was randomized in a double-blind manner to either receive oxytocin or the placebo first (neither the researcher nor the participant knew the group assignments). At the start of day 1, the participants were asked to self-administer the spray containing either oxytocin or the placebo while being monitored by a clinician under supervision of the project coordinator. On day 2, each participant self-administered the spray they had not received on day 1 according to the cross-over design.

### Visual probe task

#### Stimuli

The photographic stimuli used for the dot probe task were obtained from a validated collection of the Korean Facial Expressions of Emotions (KOFEE) [39]. The photos consisted of a pool of adult facial expressions that might provoke emotional responses. The pool was based on Ekman and Friesen's Facial Action Coding System [40]. Actors and actresses for the photos were introduced to different emotions with characteristic facial muscle contractions and were requested to perform facial muscle movements according to the directed facial actions for each emotion while the photographs were obtained. Our candidate negative emotions for this study were disgust and anger, as these emotions result in quicker detection of attentional bias and avoidance [41].

Our pool of target stimuli consisted of 60 images of adults showing 15 positive (happy) or 30 negative (15 angry and 15 disgust) expressions, with each adult having a corresponding picture showing a neutral expression (15 non-target stimuli). The target stimuli were paired with matched non-target stimuli.

#### Design and procedure

Attentional biases were assessed using a visual probe detection task [42] displaying pairs of pictures [target (emotional) and non-target (neutral)] [43]. Participants were shown a centrally positioned fixation cross for 750 ms, which was replaced by target (i.e., emotional) and non-target (i.e., neutral) pictures for 1,000 ms, following the paradigm of Mansell et al. [44].

As the two images disappeared from the screen, one of two probes (‘:’ or ‘..’) appeared where used to be the center of one of the two pictures. The participants were required to press one of two buttons on a keyboard (marked with white stickers) that corresponds to the probe (Q for ‘:’ and Z for ‘..’). When a response was made, the probe disappeared and the next trial started. Participants were advised to identify the probe as quickly and accurately as possible. The target stimuli positions and the probe positions were balanced across trials, so each appeared in either location (right or left; probe behind the target or non-target) with equal frequency, following the paradigm of Mogg and Bradley [45]. After a response was made, the probe disappeared, and the next trial started immediately.

Attention during the visual probe detection task was measured as the time to respond to the probe. The assumption is that the time to respond to the probe is faster if attention was already allocated to the spatial location where the probe appeared. Faster responses to congruent trials (when the probe emerges at the threatening stimulus location) compared to incongruent trials (when the probe emerges at the neutral stimulus location) were interpreted as vigilance to a threat.

These tests were comprised of 10 trials in each of six conditions: three target stimuli (happy, angry, and disgust) and two probe locations (the location of the target stimuli or non-target stimuli), for a total of 60 trials, repeated twice. Test trials were presented in a new random order for each participant. The dot-probe task was presented on E-prime ver. 2 (Psychology Software Tools, Inc., Pittsburgh, PA, USA).

### Objective psychological measures

#### The Eating Disorder Examination (EDE) interview [46], [47]

The Korean version of the clinician rating interview of the EDE [46], [47] was conducted with the patients with AN to establish the degree of eating psychopathology. The Korean version of the EDE has good demonstrated inter-rater reliability (Pearson's correlation coefficients  = 0.98–1.0) and internal consistency (Cronbach's alpha  = 0.72–0.89) for variables [47].

### Self-report measures

#### Eating Disorder Examination self-report version Questionnaire (EDE-Q) [48]

The EDE-Q assesses the main behavioral features of an eating disorder over the past 28 days. The questionnaire consists of 36 items on a 7-point forced choice rating scale. It measures weight, shape, eating concerns, and dietary restraint. We used the standardized Korean version of the 12th edition of the EDE-Q, which has high internal consistency and good 2 week reliability.

#### State-Trait Anger Expression Inventory (STAXI) [49]

The STAXI is a 44-item self-report questionnaire that is divided into seven scales. It measures the current intensity of anger (State Anger) and disposition toward anger as a personality trait (Trait Anger) and the tendency to express anger when criticized (Angry Reaction). The other scales measure the frequency with which provoked anger is suppressed (Anger-Suppression), the frequency of the expression of anger toward other people or objects (Anger-Expression), and the frequency at which the expression of anger is controlled (Anger-Control). The Korean version of the STAXI was used in this study [50] and has good reliability.

#### Autism-Spectrum Quotient (AQ) [51]

The AQ is a 50-item, self-administered questionnaire that assesses the degree to which adults with normal intelligence have autism spectrum traits. It has five sub-scales, including social skill, attention switching, attention to detail, communication, and imagination.

#### Other measurements

Depression and anxiety were assessed in each subject using the standardized Korean versions of the Beck Depression Inventory (BDI) [52] and the Spielberger State and Trait Anxiety Inventory (STAI) [53], respectively.

### Procedures

The participants were tested in a private room at Seoul Paik Hospital. They were instructed to abstain from alcohol and caffeine on the day of drug administration and food and drink (other than water) for 2 hours before drug administration. Upon arrival, the participants completed baseline measures of physical symptoms, including abdominal, neurological, dermatological, and cardiac symptoms. Subsequently, oxytocin or the placebo was self-administered by inhalation of the spray into each nostril. The order of oxytocin and placebo administration was randomized using Microsoft Excel in this double-blind procedure. The participants completed the self-report measures to assess their psychological state during the 45 minutes after administration of each spray. At the end of the study period, each participant completed follow-up measures for adverse physical symptoms.

### Statistical analysis

We followed the standard analysis procedure used for this task [54]. Only the trial reaction times (RT) for correct responses were included. The correct response rates were 96.1% for the AN group and 97.7% for the healthy control (HC) group. Mean RTs were calculated for each participant. Outliers were removed by excluding detection latencies that were beyond two standard deviations from their mean (i.e. from each individual's mean RTs across all stimuli).

Attentional bias scores were calculated for each matched trial type (happy-neutral, angry-neutral, disgust-neutral) as PN − PE, where PN is the mean RT for probes replacing neutral stimuli, and PE is the mean RT for probes replacing emotional target stimuli. Therefore, positive scores suggest increased attention towards the emotional/target stimuli, whereas negative scores indicate attention away from emotional/target stimuli. A score of 0 suggests no bias towards any stimuli.

The attentional responses to shape stimuli were investigated via a series of initial 2 (group: AN and HC) ×2 (drug: oxytocin and placebo) ×3 (valence: happy, angry, disgust) repeated-measures analyses of variance (ANOVAs). Post-hoc analyses were conducted through paired *t*-test comparisons for each group. Greenhouse–Geisser corrections were applied on the assumption that sphericity was violated. Data are shown as mean and with the effect size (ES) if appropriate [Cohen's *d* for t-tests; partial eta square (Δη^2^) for ANOVA]. The estimation of ES was based on Cohen's *d*: described as negligible ( = 0 and <0.15), small (≥0.15 and <0.40), medium (≥0.40 and <0.75), large (≥0.75 and <1.10), very large (≥1.10 and <1.45) and huge (>1.45) (Cohen, 1992). We defined the drug effect as the difference in the RT score between the administration of the placebo and oxytocin (drug effect  =  RT_placebo_ − RT_oxytocin_). Pearson's correlations were used to investigate the relationship between the drug effect on attentional bias and psychological variables. Analyses were performed using SPSS 19.0 (SPSS Inc., Chicago, IL, USA), and the two-tailed p-value was 0.05.

## Results

### Demographic and group characteristics

The demographic and the group characteristics of the participants are shown in Table 1. The mean age and IQ were similar between the AN and the control groups. As expected, significant differences were observed in body mass index (BMI, kg/m^2^) and the EDE-Q scales. The AN group also scored significantly higher on the BDI, STAI, and the Social Skill, Attention, and Communication subscales and the total score on the AQ compared to those in the HC group. The EDE interview scores of the AN group were 5.07±6.04 for the restraint subscale, 3.73±4.41 for the eating concern subscale, 4.35±8.33 for the weight concern subscale, and 3.85±3.10 for the shape concern subscale.

**Table 1:** Demographic and group characteristics.

	AN (*n* = 31)	Control (*n* = 33)	*t*	*df*	*p*
Age	23.10 (9.35)	22.18 (2.14)	−0.536	32.87	0.596
Intelligence	109.27 (14.01)	114.62 (9.05)	1.788	48.51	0.080
BMI	15.15 (2.51)	20.91 (2.22)	9.819^***^	62	<0.001
EDE-Q					
Restraint	2.50 (1.89)	0.79 (0.86)	−4.558^***^	39.31	<0.001
Eating Concern	2.10 (1.80)	0.55 (0.76)	−4.388^***^	38.14	<0.001
Weight Concern	2.63 (1.61)	1.46 (1.13)	−3.330^**^	51.06	0.002
Shape Concern	3.01 (1.56)	2.14 (1.31)	−2.435^*^	62	0.018
Global	2.56 (1.56)	1.23 (0.89)	−4.120^***^	44.91	<0.001
AQ					
Social Skill	3.94 (1.93)	2.67 (1.81)	−2.710^**^	62	0.009
Attention Switching	4.52 (1.93)	4.21 (1.36)	−0.724	53.67	0.472
Attention to Detail	5.03 (2.23)	3.97 (2.08)	−1.971	62	0.053
Communication	2.73 (1.82)	1.58 (1.35)	−2.848^**^	53.17	0.006
Imagination	2.29 (1.88)	2.12 (1.50)	−0.399	62	0.691
Total	18.60 (5.33)	14.55 (4.28)	−3.344^**^	62	0.001
BDI	24.53 (14.01)	7.38 (6.89)	−6.057^***^	41.64	<0.001
STAI					
State	58.47 (13.13)	43.28 (11.25)	−4.899^***^	62	<0.001
Trait	57.53 (13.46)	43.75 (11.33)	−4.371^***^	62	<0.001
STAXI					
Anger_state	16.13 (10.10)	11.66 (5.01)	−2.189^*^	41.87	0.034
Anger_trait	21.20 (7.76)	18.22 (5.96)	−1.703	60	0.094
Anger_suppression	17.10 (4.77)	17.97 (4.31)	0.753	60	0.455
Anger_expression	16.17 (3.85)	15.22 (3.85)	−0.969	60	0.337
Anger_control	17.83 (5.37)	19.72 (4.76)	1.465	60	0.148

AN, anorexia nervosa; BMI, body mass index; AQ, autism-spectrum quotient; STAI: Spielberger State-Trait Anxiety Inventory; STAXI, State and the Trait Anger Expression Inventory.

Data are shown as mean (standard deviation). *p<0.05, **p<0.01, ***p<0.001.

### Attentional bias for emotional faces using the visual attention task

A significant group x drug x emotion interaction effect (*F*(2,124)  = 3.568, *p* = 0.031, Δ*η^2^*  = 0.057) was observed in the three-way group (AN vs. HC) × drug (oxytocin vs. placebo) × emotion (happy, angry, disgust) repeated-measures ANOVA.

The two-way group (AN vs. HC) × drug (oxytocin vs. placebo) repeated-measures ANOVA identified a group × drug interaction effect (*F*(1,62)  = 4.988, *p* = 0.029, Δ*η^2^*  = 0.078).

The attentional bias scores across conditions in each group are shown in Table 2.

**Table 2:** Attentional bias scores in response to happy, angry, or disgusted faces from the visual dot probe task in either the oxytocin or placebo condition in patients with anorexia nervosa and healthy controls.

	AN (n = 31)					HC (n = 33)				
Emotional valence	Placebo	Oxytocin	*t* (*df* = 30)	*p*	Cohen's *d*	Placebo	Oxytocin	*t* (*df* = 32)	*p*	Cohen's *d*
Happy	−0.66 (84.92)	3.16 (73.15)	−0.168	0.868	−0.048	1.05 (66.34)	1.94 (61.03)	−0.057	0.955	−0.014
Angry	−38.27 (104.70)	48.00 (117.90)	−2.847^**^	0.008	−0.774	25.38 (97.73)	−3.29 (77.66)	1.218	0.233	0.325
Disgust	24.34 (100.73)	−16.38 (94.05)	1.602	0.120	0.408	21.90 (60.48)	−38.26 (79.99)	3.138^**^	0.004	0.848

AN, anorexia nervosa; HC, healthy control.

Data are shown as mean (standard deviation) *p<0.05, **p<0.01.

The two-way group (AN vs. HC) × drug (oxytocin vs. placebo) repeated-measures ANOVA showed that there was neither a main effect of drug (*F*(1,62)  = 0.028, *p* = 0.868, Δ*η^2^*  = 0.000) nor a group × drug interaction effect in response to happy stimuli (*F*(1,62)  = 0.009, *p* = 0.925, Δ*η^2^*  = 0.000).

The two-way group (AN vs. HC) × drug (oxytocin vs. placebo) repeated-measures ANOVA showed that there was a group × drug interaction effect (*F*(1,62)  = 8.970, *p* = 0.004, Δ*η^2^*  = 0.130) in response to the angry stimuli. Post-hoc tests showed avoidance of angry faces in the AN group and vigilance in the HC group with a moderate to large difference between the AN and HC groups under the placebo condition (*t*(62)  = 2.510, *p* = 0.015, *d* = −0.633). Both of these information processing responses were moderated by oxytocin and produced a large increase in vigilance in the AN group (*t*(30)  = −2.847, *p* = 0.008, *d* = 0.785) but a tendency to decrease with a small ES in attention in the HC group (*t*(32)  = 1.218, *p* = 0.233, *d* = 0.219).

**Figure 1:**
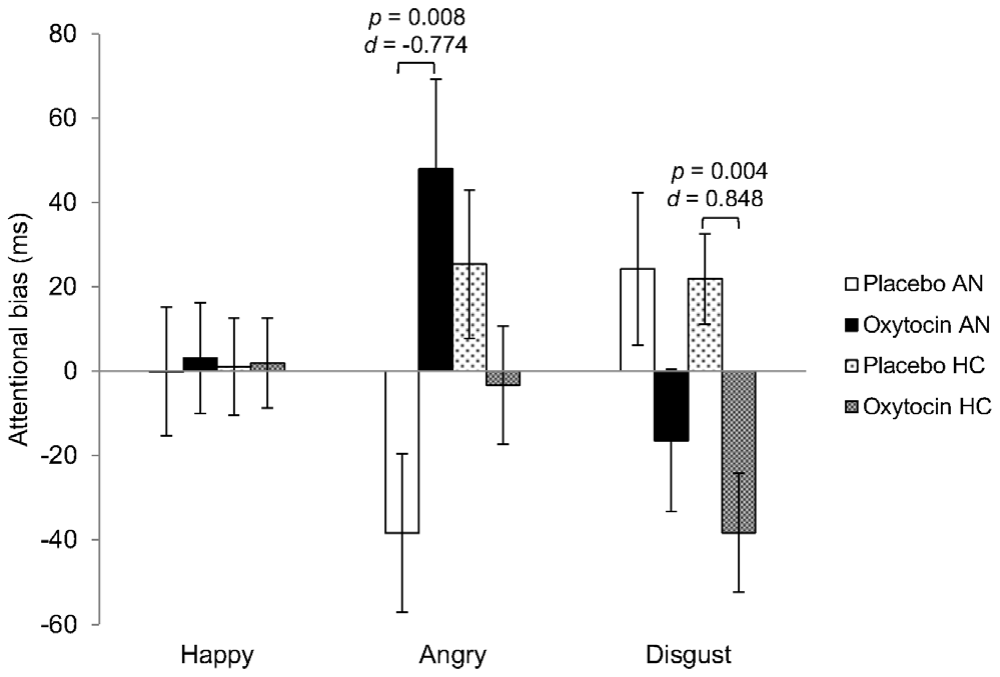
The effect of oxytocin on attentional bias to facial stimuli with happy, angry, or disgusted expressions in patients with AN and HC. Data are mean and standard error (AN, anorexia nervosa; HC, healthy control). Avoidance of angry faces was observed in the AN group and vigilance to them was observed in the HC group under the placebo condition; both of these responses were moderated by oxytocin. In particular, vigilance to anger significantly increased in the AN group (AN: *t*(30)  = −2.847, *p* = 0.008, *d* = −0.774; HC: *t*(32)  = 1.218, *p* = 0.233, *d* = 0.325). Attentional biases to the disgust stimuli were observed in both groups under the placebo condition. The attentional biases to disgust decreased under the oxytocin condition (AN: *t*(30)  = 1.602, *p* = 0.120, *d* = 0.418; HC: *t*(32)  = 3.138, *p* = 0.004, *d* = 0.848). No marked attention to happy faces was observed in either the placebo or oxytocin conditions.

The two-way group (AN vs. HC) × drug (oxytocin vs. placebo) repeated-measures ANOVA showed a main effect of drug (*F*(1,62)  = 10.120, *p* = 0.002, Δ*η^2^*  = 0.142) with no group × drug interaction effect (*F*(1,62)  = 0.375, *p* = 0.542, Δ*η* ^2^  = 0.006) in response to the disgust stimuli. Post-hoc tests showed an attentional bias to the disgust stimuli in both the AN and HC groups under the placebo condition (*t*(62)  = 3.208, *p* = 0.002, *d* = 0.597). Attentional bias was reduced under the oxytocin condition in both groups with a small effect in the AN group (*t*(30)  = 1.602, *p* = 0.120, *d* = 0.418) and a moderate/large effect in the HC group (*t*(32)  = 3.138, *p* = 0.004, *d* = 0.555). The attentional bias scores across conditions in each group are shown in Figure 1.

### Association between attentional biases and clinical variables

The reduced vigilance to angry stimuli in the HC group was positively correlated with STAXI subscales of anger expression in the oxytocin condition (*r* = 0.415, *p* = 0.023, *n* = 33) i.e., individuals with higher anger expression showed a greater reduction in vigilance with oxytocin. There was no association between STAXI subscales and the oxytocin effect on angry stimuli in patients with AN. No association was observed between the drug effect and aspects of psychopathology typically associated with eating disorders (EDE-Q), depression (BDI), state and trait anxiety (STAI), autism traits (AQ) in either the AN or HC groups. No association between BMI or weight and the oxytocin effect were observed in either the AN group (BMI/weight: *p* = 0.824/0.837 for happy faces, *p* = 0.164/0.353 for angry faces, *p* = 0.844/0.741 for disgust faces) or in the HC group (*p* = 0.646/0.964 for happy faces, *p* = 0.842/0.683 for angry faces, *p* = 0.991/0.898 for disgust faces).

## Discussion

The aim of this study was to examine whether oxytocin had an impact on social attentional processes in patients with AN. Unlike our prediction, avoidance of, rather than vigilance to, angry faces was observed in the patients with AN under the placebo condition and vigilance to anger was observed in the HC. Vigilance to disgust was observed in both patients with AN and the HC group. Happy (smiling) faces produced no attentional bias in patients with AN or the HC group. Our second hypothesis was confirmed, in part, as oxytocin attenuated the selective attention towards disgust in both the patients with AN and the HC group. In contrast, oxytocin converted an avoidant response to anger into vigilance in patients with AN but reduced the vigilant response to anger in the HC group. Oxytocin did not change the lack of attention to happy (smiling) faces in either the AN or HC groups.

Our result that smiling faces did not produce an attentional bias in either the AN or HC groups was consistent with other findings in Caucasian patients with AN [15]. However, the same laboratory found that faces depicting warmth and acceptance using the stimulus set of Dandeneau, Baldwin, Baccus, Sakellaropoulo, and Pruessner produced attentional biases (avoidance in AN and vigilance in HC) [18]. Ekman [55] described many different types of smiles and distinguished the Duchenne smile [56], which communicates positive emotions, from other types of smiles, which may be aversive and processed as a threat by some individuals [57]. Thus, a more nuanced approach to stimulus sets of faces depicting social reward may be needed.

In the oxytocin condition, the attentional bias was away from disgusted faces in both groups with a larger effect in the HC group. To our knowledge no previous study has evaluated the impact of oxytocin on recognition of the disgust emotion in humans. The results are consistent with another study, which found that oxytocin decreases attention toward negative faces (i.e., sad) in healthy people [58].

The avoidance response to anger expression in patients with AN and a vigilant response in the HC group under the placebo condition differed from a study that used “rejecting” facial expressions in which the opposite pattern was found, i.e., vigilance to the rejecting stimuli in patients with AN [18]. One interpretation of this difference is that rejecting faces have different implications than angry ones. Interestingly, when given oxytocin, patients with AN became vigilant to these cues, whereas vigilance in the healthy group diminished. Moreover, the response to oxytocin appeared to be moderated by the trait of higher anger expression. The decreased in attentional bias to angry stimuli in the HC group was associated with higher anger expression, whereas no association between increased vigilance to the angry face and STAXI subscales was observed in patients with AN. These findings suggest that patients with AN may use different strategies and circuits to regulate anger from those of healthy people. It has been suggested that patients with AN may have difficulties with anger, perceiving it as “toxic” and unacceptable, which may lead to anger suppression [59]. The pattern of directing anger and hostility inwardly in patients with AN is supported by previous studies [60]–[62]. Women with AN have lower levels of anger expression than those with bulimia nervosa [63], and increased difficulties coping with anger and frustration are found in those with AN [64]. The vigilant reaction of people with AN to anger cues under the oxytocin condition was more like the HC group in the placebo condition. This is coherent with the idea that the avoidance of emotional processing through self-starvation is a maladaptive coping strategy [65].

These findings raise the possibility that oxytocin treatment may moderate some of these social difficulties and possibly improve the outcome. Resistance to treatment is associated with less activation in social cognition circuits within the brain [66]. The vicious cycle of maintenance in patients with AN is self-perpetuated either through starvation or through feelings of low self-worth and/or fear of rejection [67]. The appropriate acknowledgment and expression of anger and frustration, through aggressive or assertive behavior, may be vital for functional social experience [68].

There are some limitations in this study that need to be considered and which may account for some of the individual variation in this study. First, a third of the patients with AN were recruited from the outpatient clinic and the others were from the inpatient ward. So the patients were at different phases of treatment. The oxytocinergic pathway may differ in the more severely affected patients undergoing refeeding, compared to that of outpatients. However, all of our participants were screened when they initially visited to the clinic and were in the active stage of the illness, regardless of their inpatient or the outpatient status. None of them were weight-recovered. This is a proof of concept study which aimed to broadly examine that oxytocin might be of benefit in the short term across different stages. Further studies should consider controlling the illness stages. Second, the doses of intranasal oxytocin were not adjusted for individual differences in BMI and therefore the underweight patients received a higher doses per weight than healthy controls. However, in our subsidiary analysis, no relationship was observed between the BMI or weight and the oxytocin effect in either the AN or healthy control groups. Further studies examining for a dose relationship may be of interest and reduce the variance. Third, three patients took fluoxetine, which may affect the oxytocin pathway, so may have increased the variance. An additional analysis found no differences in the response of this subgroup to the non medicated patients. Finally, we did not measure other individual factors that are known to impact attention bias. For example, individuals with a short 5-hydroxytryptamine allele have been found to be more vigilant towards threat [69]. Additionally, the experience of childhood care, personality, and interpersonal context are known to influence the effects of oxytocin [70].

In conclusion, patients with AN showed an avoidance of angry faces whereas healthy women were vigilant to anger in the placebo condition. This response in the AN group shifted from avoidance to vigilance following oxytocin administration but there was a trend for the healthy women to be less vigilant to angry faces. Patients with AN showed vigilance to faces of disgust similar to the healthy women and this was moderated with oxytocin in both groups reaching a significant effect in healthy women. Neither group had any attentional bias to happy faces under either the oxytocin or control conditions. These findings suggest that further evaluation of oxytocin nasal spray as a treatment to improve emotional processing and social communication particularly in relationship to anger may be of value in patients with AN.

## References

[1] Allison S, Warin M, Bastiampillai T [published online ahead of print August 22, 2013] Anorexia nervosa and social contagion: Clinical implications. Aust N Z J Psychiatry doi: 10.1177/0004867413502092 10.1177/0004867413502092 23969627

[2] Sharpe H, Naumann U, Treasure J, Schmidt U (2013) Is fat talking a causal risk factor for body dissatisfaction? A systematic review and meta-analysis. Int J Eat Disord 46: 643–652. 23818118 10.1002/eat.22151

[3] Menzel JE, Schaefer LM, Burke NL, Mayhew LL, Brannick MT, et al (2010) Appearance-related teasing, body dissatisfaction, and disordered eating: A meta-analysis. Body Image 7: 261–270. 20655287 10.1016/j.bodyim.2010.05.004

[4] Gillberg CL (1992) Autism and autistic-like conditions - subclasses among disorders of empathy. J Child Psychol Psyc 33: 813–842. 10.1111/j.1469-7610.1992.tb01959.x 1634591

[5] Krug I, Penelo E, Fernandez-Aranda F, Anderluh M, Bellodi L, et al (2012) Low social interactions in eating disorder patients in childhood and adulthood: A multi-centre European case control study. J Health Psychol 18: 26–37. 22491496 10.1177/1359105311435946

[6] Allen KL, Byrne SM, Forbes D, Oddy WH (2009) Risk factors for full- and partial-syndrome early adolescent eating disorders: A population-based pregnancy cohort study. J Am Acad Child Adolesc Psychiatry 48: 800–809. 19564799 10.1097/CHI.0b013e3181a8136d

[7] Treasure J, Corfield F, Cardi V (2012) A Three-phase Model of the Social Emotional Functioning in Eating Disorders. Eur Eat Disord Rev 20: 431–438. 22539368 10.1002/erv.2181

[8] McKnight R, Boughton N (2009) A patient's journey Anorexia nervosa. BMJ 339: b3800. 19778976 10.1136/bmj.b3800

[9] Cacioppo JT, Decety J (2011) Social neuroscience: challenges and opportunities in the study of complex behavior. Ann N Y Acad Sci 1224: 162–173. 21251011 10.1111/j.1749-6632.2010.05858.x PMC3098127

[10] Michopoulos V, Higgins M, Toufexis D, Wilson ME (2012) Social subordination produces distinct stress-related phenotypes in female rhesus monkeys. Psychoneuroendocrinology 37: 1071–1085. 22244748 10.1016/j.psyneuen.2011.12.004 PMC3358530

[11] Arcelus J, Haslam M, Farrow C, Meyer C (2013) The role of interpersonal functioning in the maintenance of eating psychopathology: A systematic review and testable model. Clinl Psychol Rev 33: 156–167. 10.1016/j.cpr.2012.10.009 23195616

[12] Oldershaw A, Hambrook D, Stahl D, Tchanturia K, Treasure J (2011) The socio-emotional processing stream in Anorexia Nervosa. Neurosci Biobehav Rev 35: 970–988. 21070808 10.1016/j.neubiorev.2010.11.001

[13] Zucker NL, Losh M, Bulik CM, LaBar KS, Piven J, et al (2007) Anorexia nervosa and autism spectrum disorders: guided investigation of social cognitive endophenotypes. Psychol Bull 133: 976–1006. 17967091 10.1037/0033-2909.133.6.976

[14] Treasure J, Corfield F, Cardi V (2012) A three-phase model of the social emotional functioning in Eating Disorders. Eur Eat Disord Rev 20: 431–438. 22539368 10.1002/erv.2181

[15] Corfield F, Cardi V, Leppanen J, Rhind C, Deriziotis S, et al.. Elicited facial expression, emotional experience and attention to infant cues in anorexia nervosa. Manuscript submitted for publication.

[16] Davies H, Schmidt U, Stahl D, Tchanturia K (2011) Evoked facial emotional expression and emotional experience in people with anorexia nervosa. Int J Eat Disord 44: 531–539. 20957704 10.1002/eat.20852

[17] Claes L, Jimenez-Murcia S, Santamaria JJ, Moussa MB, Sanchez I, et al (2012) The facial and subjective emotional reaction in response to a video game designed to train emotional regulation (playmancer). Eur Eat Disord Rev 20: 484–489. 23097370 10.1002/erv.2212

[18] Cardi V, Matteo RD, Corfield F, Treasure J (2012) Social reward and rejection sensitivity in Eating Disorders: an investigation of attentional bias and early experiences. World J Biol Psychiatry 14: 622–633. 22424288 10.3109/15622975.2012.665479

[19] Prehn K, Kazzer P, Lischke A, Heinrichs M, Herpertz SC (2013) Effects of intranasal oxytocin on pupil dilation indicate increased salience of socioaffective stimuli. Psychophysiology 50: 528–537. 23551070 10.1111/psyp.12042

[20] Leknes S, Wessberg J, Ellingsen DM, Chelnokova O, Olausson H, et al (2013) Oxytocin enhances pupil dilation and sensitivity to ‘hidden’ emotional expressions. Soc Cognit Affect Neurosci 8: 741–749. 22648957 10.1093/scan/nss062 PMC3791062

[21] Parr LA, Modi M, Siebert E, Young LJ (2013) Intranasal oxytocin selectively attenuates rhesus monkeys' attention to negative facial expressions. Psychoneuroendocrinology 38: 1748–1756. 23490074 10.1016/j.psyneuen.2013.02.011 PMC3743934

[22] Domes G, Steiner A, Porges SW, Heinrichs M (2013) Oxytocin differentially modulates eye gaze to naturalistic social signals of happiness and anger. Psychoneuroendocrinology 38: 1198–1202. 23117026 10.1016/j.psyneuen.2012.10.002

[23] Lischke A, Gamer M, Berger C, Grossmann A, Hauenstein K, et al (2012) Oxytocin increases amygdala reactivity to threatening scenes in females. Psychoneuroendocrinology 37: 1431–1438. 22365820 10.1016/j.psyneuen.2012.01.011

[24] Bertsch K, Gamer M, Schmidt B, Schmidinger I, Walther S, et al (2013) Oxytocin and Reduction of Social Threat Hypersensitivity in Women With Borderline Personality Disorder. Am J Psychiatry 170: 1169–1177. 23982273 10.1176/appi.ajp.2013.13020263

[25] Brune M, Ebert A, Kolb M, Tas C, Edel MA, et al. [published online ahead of print August 16, 2013] Oxytocin influences avoidant reactions to social threat in adults with borderline personality disorder. Hum Psychopharmacol doi: 10.1002/hup.2343 10.1002/hup.2343 23950057

[26] Tollenaar MS, Chatzimanoli M, van der Wee NJ, Putman P (2013) Enhanced orienting of attention in response to emotional gaze cues after oxytocin administration in healthy young men. Psychoneuroendocrinology 38: 1792–1802. 10.1016/j.psyneuen.2013.02.018 23562249

[27] Domes G, Sibold M, Schulze L, Lischke A, Herpertz SC (2013) Intranasal oxytocin increases covert attention to positive social cues. Psychol Med 43: 1747–1753. 23146328 10.1017/S0033291712002565

[28] Guastella AJ, Carson DS, Dadds MR, Mitchell PB, Cox RE (2009) Does oxytocin influence the early detection of angry and happy faces? Psychoneuroendocrinology 34: 220–225. 18848401 10.1016/j.psyneuen.2008.09.001

[29] Kim YR, Oh SM, Corfield F, Jeong DW, Jang EY, et al.. (In press) Intranasal oxytocin lessens the attentional bias to adult negative faces: A double blind within-subject experiment. Psychiatry Investig. 10.4306/pi.2014.11.2.160 PMC4023090 24843371

[30] Shahrestani S, Kemp AH, Guastella AJ (2013) The Impact of a Single Administration of Intranasal Oxytocin on the Recognition of Basic Emotions in Humans: A Meta-Analysis. Neuropsychopharmacology 38: 1929–1936. 23575742 10.1038/npp.2013.86 PMC3746698

[31] Maguire S, O'Dell A, Touyz L, Russell J (2013) Oxytocin and anorexia nervosa: a review of the emerging literature. Eur Eat Disord Rev 21: 475–8. 24115458 10.1002/erv.2252

[32] Chiodera P, Volpi R, Capretti L, Marchesi C, d'Amato L, et al (1991) Effect of estrogen or insulin-induced hypoglycemia on plasma oxytocin levels in bulimia and anorexia nervosa. Metabolism 40: 1226–1230. 1943752 10.1016/0026-0495(91)90220-q

[33] Demitrack MA, Lesem MD, Listwak SJ, Brandt HA, Jimerson DC, et al (1990) CSF oxytocin in anorexia nervosa and bulimia nervosa: clinical and pathophysiologic considerations. Am J Psychiatry 147: 882–886. 2356873 10.1176/ajp.147.7.882

[34] Lawson EA, Donoho DA, Blum JI, Meenaghan EM, Misra M, et al (2011) Decreased nocturnal oxytocin levels in anorexia nervosa are associated with low bone mineral density and fat mass. J Clin Psychiatry 72: 1546–1551. 21903023 10.4088/JCP.10m06617 PMC3731046

[35] Lawson EA, Holsen LM, Santin M, Meenaghan E, Eddy KT, et al (2012) Oxytocin secretion is associated with severity of disordered eating psychopathology and insular cortex hypoactivation in anorexia nervosa. J Clin Endocrinol Metab 97: E1898–1908. 22872688 10.1210/jc.2012-1702 PMC3674290

[36] First MB, Spitzer RL, Gibbon M, Williams JBW (2002) Structured Clinical Interview for DSM-IV-TR Axis I Disorders-Patient Edition (SCID-I/P). New York: Biometrics Research Department, New York State Psychiatric Institute.

[37] Murphy SE, Yiend J, Lester KJ, Cowen PJ, Harmer CJ (2009) Short-term serotonergic but not noradrenergic antidepressant administration reduces attentional vigilance to threat in healthy volunteers. Int J Neuropsychopharmacol 12: 169–179. 18752726 10.1017/S1461145708009164

[38] MacDonald E, Dadds M, Brennan J, Williams K, Levy F, et al (2011) A review of safety, side-effects and subjective reactions to intranasal oxytocin in human research. Psychoneuroendocrinology 36: 1114–1126. 21429671 10.1016/j.psyneuen.2011.02.015

[39] Park JY, Oh JM, Kim SY, Lee MK, Lee CR, et al.. (2011) Korean Facial Expressions of Emotion (KOFEE). Seoul, South Korea. Section of Affect & Neuroscience, Institute of Behavioral Science in Medicine, Yonsei University College of Medicine.

[40] Ekman P, Friesen WV (1978) Facial action coding system: A technique for the measurement of facial movement. Palo Alto, CA: Consulting Psychologists Press.

[41] Gilboa-Schechtman E, Foa EB, Amir N (1999) Attentional biases for facial expressions in social phobia: The face-in-the-crowd paradigm. Cognition Emotion 13: 305–318.

[42] Posner MI, Snyder CRR, Davidson BJ (1980) Attention and the detection of signals J Exp Psychol Gen. 109: 160–174. 7381367

[43] Macleod C, Mathews A, Tata P (1986) Attentional bias in emotional disorders. J Abnorm Psychol 95: 15–20. 3700842 10.1037//0021-843x.95.1.15

[44] Mansell W, Clark DM, Ehlers A, Chen YP (1999) Social anxiety and attention away from emotional faces. Cognition Emotion 13: 673–690.

[45] Mogg K, Bradley BP (1998) A cognitive-motivational analysis of anxiety. Behav Res Ther 36: 809–848. 9701859 10.1016/s0005-7967(98)00063-1

[46] Heo S, Lee M, Choi Y, Sohn C, Lee H (2004) Reliability and factor analysis of the Korean version of Eating Disorder Examination. J Korean Soc Study Obes 13: 42–52.

[47] Fairburn CG, Cooper Z (1993) The Eating Disorder Examination (12th ed.); Fairburn CG, Wilson GT, editors. New York: Guilford.

[48] Fairburn C, Beglin S (1994) Assessment of eating disorders—Interview or self-report questionnaire. Int J Eat Disord 16: 363–370. 7866415

[49] Spielberger CD (1991) State-Trait Anger Expression Inventory. Odessa, FL: Psychological Assessment Resources.

[50] Chon KK (1996) Development of the Korean State-Trait Anger Expression Inventory. Kor J Clin Psychol 3: 53–69.

[51] Baron-Cohen S, Wheelwright S, Skinner R, Martin J, Clubley E (2001) The Autism-Spectrum Quotient (AQ): Evidence from Asperger syndrome/high-functioning autism, males and females, scientists and mathematicians. J Autism Dev Disord 31: 5–17. 11439754 10.1023/a:1005653411471

[52] Beck AT, Ward CH, Mendelson M, Mock JE, Erbaugh J (1961) An inventory for measuring depression. Arch Gen Psychiatry 4: 561–571. 13688369 10.1001/archpsyc.1961.01710120031004

[53] Spielberger C, Gorsuch R, Lushene R, Vagg P, Facobs G (1983) Manual for the State-Trait Anxiety Inventory, STAI (Form Y). Palo Alto: Consulting Psychologists Press.

[54] Bradley BP, Hogg K, White J, Groom C, de Bono J (1999) Attentional bias for emotional faces in generalized anxiety disorder. Br J Clin Psychol 38: 267–278. 10532148 10.1348/014466599162845

[55] Ekman P (1992) Facial expressions of emotion - new findings, new questions. Psychol Sci 3: 34–38.

[56] Ekman P, Friesen WV, Davidson RJ (1990) The Duchenne smile - emotional expression and brain physiology. 2. J Pers Soc Psychol 58: 342–353. 2319446

[57] Ekman P (1992) Facial expressions of emotion - an old controversy and new findings Philos Trans R Soc Lond B Biol Sci. 335: 63–69. 10.1098/rstb.1992.0008 1348139

[58] Ellenbogen MA, Linnen AM, Grumet R, Cardoso C, Joober R (2012) The acute effects of intranasal oxytocin on automatic and effortful attentional shifting to emotional faces. Psychophysiology 49: 128–137. 22092248 10.1111/j.1469-8986.2011.01278.x

[59] Harrison A, Genders R, Davies H, Treasure J, Tchanturia K (2011) Experimental measurement of the regulation of anger and aggression in women with anorexia nervosa. Clin Psychol Psychother 18: 445–452. 20859934 10.1002/cpp.726

[60] Geller J, Cockell SJ, Goldner EM (2000) Inhibited expression of negative emotions and interpersonal orientation in anorexia nervosa. Int J Eat Disord 28: 8–19. 10800009 10.1002/1098-108x(200007)28:1<8::aid-eat2>3.0.co;2-u

[61] Oldershaw A, Hambrook D, Tchanturia K, Treasure J, Schmidt U (2010) Emotional Theory of Mind and Emotional Awareness in Recovered Anorexia Nervosa Patients. Psychosom Med 72: 73–79. 19995886 10.1097/PSY.0b013e3181c6c7ca

[62] Miotto P, Pollini B, Restaneo A, Favaretto G, Preti A (2008) Aggressiveness, anger, and hostility in eating disorders. Compr Psychiatry 49: 364–373. 18555057 10.1016/j.comppsych.2008.01.004

[63] Fassino S, Daga GA, Piero A, Leombruni P, Rovera GG (2001) Anger and personality in eating disorders. J Psychosom Res 51: 757–764. 11750298 10.1016/s0022-3999(01)00280-x

[64] Krug I, Bulik CM, Vall-Llovera ON, Granero R, Aguera Z, et al (2008) Anger expression in eating disorders: Clinical, psychopathological and personality correlates. Psychiatry Res 161: 195–205. 18838172 10.1016/j.psychres.2007.10.003

[65] Schmidt U, Treasure J (2006) Anorexia nervosa: Valued and visible. A cognitive-interpersonal maintenance model and its implications for research and practice. Br J Clin Psychol 45: 343–366. 17147101 10.1348/014466505x53902

[66] Schulte-Ruther M, Mainz V, Fink GR, Herpertz-Dahlmann B, Konrad K (2012) Theory of mind and the brain in anorexia nervosa: relation to treatment outcome. J Am Acad Child Adolesc Psychiatry 51 : 832–841 e811. 10.1016/j.jaac.2012.06.007 22840554

[67] Aspen V, Darcy AM, Lock J (2013) A review of attention biases in women with eating disorders. Cognition Emotion 27: 820–838. 23228135 10.1080/02699931.2012.749777 PMC3610839

[68] Ochsner KN, Gross JJ (2008) Cognitive emotion regulation: Insights from social cognitive and affective neuroscience. Curr Dir Psychol Sci 17: 153–158. 25425765 10.1111/j.1467-8721.2008.00566.x PMC4241349

[69] Pergamin-Hight L, Bakermans-Kranenburg MJ, van Ijzendoorn MH, Bar-Haim Y (2012) Variations in the promoter region of the serotonin transporter gene and biased attention for emotional information: A meta-analysis. Biol Psychiatry 71: 373–379. 22138391 10.1016/j.biopsych.2011.10.030

[70] Bakermans-Kranenburg MJ, van IJMH (2013) Sniffing around oxytocin: review and meta-analyses of trials in healthy and clinical groups with implications for pharmacotherapy. Transl Psychiatry 3: e258. 23695233 10.1038/tp.2013.34 PMC3669921

